# New Cembrane Diterpenoids from a Hainan Soft Coral *Sinularia* sp

**DOI:** 10.3390/md10092023

**Published:** 2012-09-18

**Authors:** Bin Yang, Xuefeng Zhou, Hui Huang, Xian-Wen Yang, Juan Liu, Xiuping Lin, Xiubao Li, Yan Peng, Yonghong Liu

**Affiliations:** 1 Key Laboratory of Marine Bio-Resources Sustainable Utilization, South China Sea Institute of Oceanology, Chinese Academy of Sciences, Guangzhou 510301, China; Email: bingo525@163.com (B.Y.); xfzhou@scsio.ac.cn (X.Z.); huanghui@scsio.ac.cn (H.H.); xwyang@scsio.ac.cn (X.-W.Y.); ljuan2010@qq.com (J.L.); xiupinglin@hotmail.com (X.L.); lixiubao@scsio.ac.cn (X.L.); py00_2006@126.com (Y.P.); 2 Guangdong Key Laboratory of Marine Materia Medica, RNAM Center for Marine Microbiology, South China Sea Institute of Oceanology, Chinese Academy of Sciences, Guangzhou 510301, China

**Keywords:** *Sinularia* sp., cembrane diterpenoids, NF-κB inhibitor

## Abstract

Five new cembrane diterpenoids, named sinuflexibilins A–E (**1**–**5**), along with nine other known diterpenoids (**6**–**14**), have been isolated from the organic extract of a Hainan soft coral *Sinularia* sp. Their structures were determined on the basis of extensive spectroscopic analyses and by comparison of their spectral data with those of related metabolites. Compound **13**, flexibilide, exhibited significant inhibitory activity of NF-κB activation using the cell-based HEK293 NF-κB luciferase reporter gene assay.

## 1. Introduction

Cembrane diterpenoids and their cyclized derivatives are the most abundant secondary metabolites of soft corals and gorgonians [1,2,3]. There is a wide range of structural complexity within this series. These cembranes represent the main chemical defense tools of animals against their natural predators such as other corals, and fishes, as well as the settlement of microorganisms [4,5]. In addition, cembranes also exhibit a wide range of biological activities including anti-inflammatory [6,7,8], and antitumor properties [9,10]. 

Genus *Sinularia* is one of the most widely distributed soft corals. It constitutes a dominant portion of the biomass in the tropical reef environment. *Sinularia* elaborates a rich harvest of secondary metabolites, including sesquiterpenes, diterpenoids, polyhydroxylated steroids, and polyamine compounds [11,12,13,14]. These metabolites were recently shown to possess a range of biological activities [15]. Cembranes are the most frequent secondary metabolites isolated from various *Sinularia* species [16,17,18].

**Figure 1:**
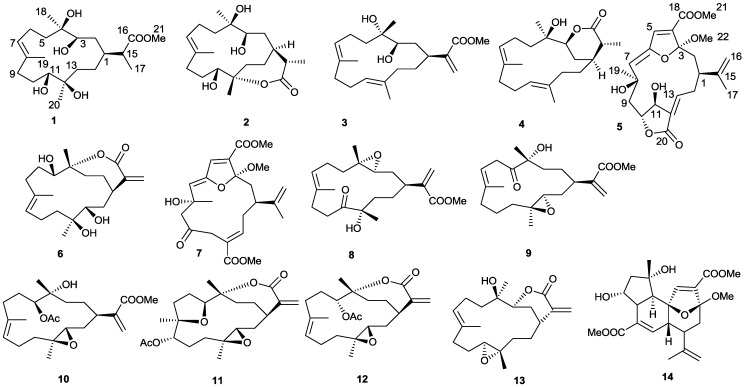
Structures of compounds **1**–**14** from *Sinularia* sp.

Nuclear factor-kappa B (NF-κB) is a protein complex that controls the transcription of DNA. NF-κB plays a key role in regulating the immune response to infection. Incorrect regulation of NF-κB has been linked to cancer, inflammatory and autoimmune diseases, septic shock, viral infection, and improper immune development [19]. Our recent investigation of bioactive natural products from a Hainan soft coral, *Sinularia* sp., has led to the isolation of five new cembranes (**1**–**5**), along with nine other known diterpenoids (**6**–**14**) (Figure 1). In this paper, we report the isolation and structural elucidation of these diterpenoids and their activities as inhibitors of NF-κB.

## 2. Results and Discussion

**Figure 2:**
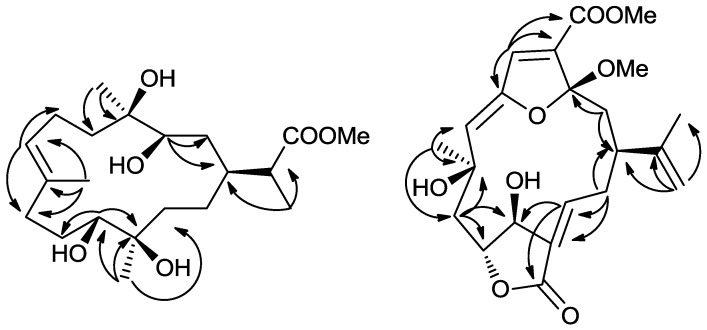
Key HMBC correlations **1** and **5**.

**Figure 3:**
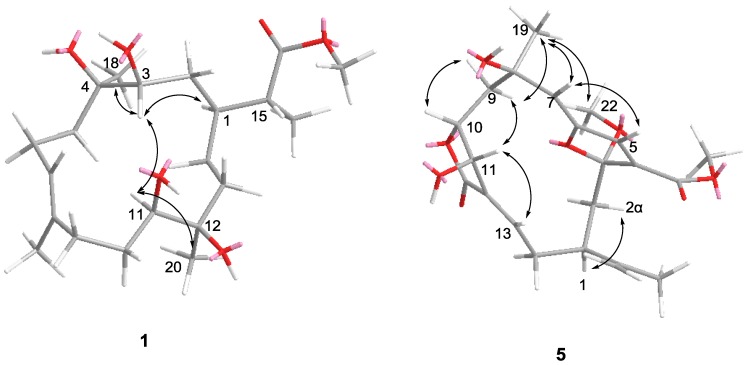
Key NOE correlations **1** and **5**.

Compound **1** was isolated as a colorless oil. The HRESIMS of **1** established its molecular formula as C_21_H_38_O_6_, indicating three unsaturations. Resonances due to olefinic carbons (*δ*_C_ 133.9, 128.7), and one carboxyl (*δ*_C_ 178.9) in the ^13^C NMR spectrum accounted for two double-bond equivalents, indicating that **1** was a monocyclic compound (Table 1). Signals for a vinyl methyl at *δ* 1.68 (3H, s), one methoxy group at *δ* 3.68 (3H, s), a methyl doublet at *δ* 1.17 (3H, d, *J* = 7.0 Hz), and two tertiary oxygenated methyl groups at *δ* 1.15 (3H, s), and 1.08 (3H, s) were observed in the ^1^H NMR spectrum (Table 2). Carbon signals at *δ* 70.6, 71.0, 75.3, and 75.7, and two proton signals at *δ* 3.52, and 3.65 indicated the presence of two secondary and two tertiary hydroxyl groups. A signal at *δ* 5.43 attributed to an olefinic proton, together with a methyl carbon signal at *δ* 17.0, indicated the *E* configuration for this double bond. These data suggested that **1** was a rearranged cembrane. Key HMBC correlations from H_3_-20 to C-13, C-12, and C-11; H-11 to C-12, C-10, and C-20; H_3_-19 to C-7, C-8, and C-9; H-7 to C-9, C-6, and C-19; H_3_-18 to C-5, C-4, and C-3; and H-13 to C-14, and C-1 established the 14-membered ring structure of **1** (Figure 2). The isopropyl acid group was found based on the HMBC correlations observed from H_3_-21 to C-1, C-15, and C-16; H-15 to C-1, C-2, C-21, and C-16. Furthermore, the methoxyl substituent was shown to be connected to position C-16 on the basis of the HMBC correlation between the oxymethyl protons (*δ*_H_ 3.68) and the carbonyl carbon (*δ*_C_ 178.9). The NMR spectra of compound **1** were almost identical with those of sinuflexibilin [20] with the exception that the *exo*-methylene proton resonances of the latter were replaced by a methyl doublet. The stereochemistry of **1** was determined on the basis of the chemical shift and NOESY spectrum (Figure 3). NOE correlations from H-3 to H-1, H-11, H_3_-18 and from H-11 to H-20 indicated that all four hydroxy groups in **1** were β-oriented and H-1, H-3, H-11, H_3_-18, and CH_3_-20 were α-oriented with respect to this ring. 

**Table 1:** ^13^C NMR (125 MHz) data for compounds **1**–**5** in CDCl_3_.

Position	1	2	3	4	5
1	38.4 CH	34.8 CH	39.4 CH	36.4 CH	40.9 CH
2	33.3 CH_2_	37.2 CH_2_	35.1 CH_2_	36.6 CH_2_	38.3 CH_2_
3	71.0 CH	74.2 CH	71.7 CH	84.3 CH	116.6 C
4	75.7 C	76.3 C	75.2 C	74.3 C	131.1 C
5	39.4 CH_2_	39.9 CH_2_	35.2 CH_2_	37.8 CH_2_	139.5 CH
6	24.1 CH_2_	23.7 CH_2_	25.3 CH_2_	23.9 CH_2_	150.0 C
7	128.7 CH	128.9 CH	124.1 CH	124.6 CH	117.2 CH
8	133.9 C	135.4 C	135.9 C	134.5 C	71.5 C
9	35.6 CH_2_	37.5 CH_2_	39.5 CH_2_	39.4 CH_2_	40.9 CH_2_
10	27.9 CH_2_	28.8 CH_2_	26.4 CH_2_	22.4 CH_2_	81.9 CH
11	70.6 CH	68.6 CH	126.1 CH	126.5 CH	75.4 CH
12	75.3 C	88.7 C	134.1 C	132.3 C	131.5 C
13	34.9 CH_2_	33.2 CH_2_	34.7 CH_2_	26.8 CH_2_	145.4 CH
14	22.2 CH_2_	26.5 CH_2_	28.2 CH_2_	30.4 CH_2_	32.5 CH_2_
15	44.5 CH	42.2 CH_2_	144.3 C	42.0 CH	147.3 C
16	178.9 C	181.5 C	168.8 C	175.1 C	112.9 CH_2_
17	15.2 CH_3_	11.0 CH_3_	124.3 CH_2_	16.3 CH_3_	18.4 CH_3_
18	23.6 CH_3_	24.0 CH_3_	23.4 CH_3_	24.8 CH_3_	162.2 C
19	17.0 CH_3_	15.8 CH_3_	16.4 CH_3_	14.1 CH_3_	30.3 CH_3_
20	24.1 CH_3_	23.1 CH_3_	15.6 CH_3_	15.2 CH_3_	168.1 C
21	51.9 CH_3_		52.0 CH_3_		51.9 CH_3_
22					50.2 CH_3_

**Table 2:** ^1^H NMR (500 MHz) data for compounds **1**–**5 **in CDCl_3_, *δ* in ppm and *J* in Hz.

Position	1	2	3	4	5
1	1.98 m	1.96 m	2.90 m	1.30 m	2.27 m
2	1.64 m	1.87 m	2.05 m	2.12 m	2.48 dd (8.0, 6.0)
	1.19 m	1.23 m	1.42 m		1.87 d (14.0)
3	3.65 d (10.5)	3.36 m	3.67 m	4.03 d (10.5)	
5	2.02 m	1.82 m	2.26 m	1.75 m	7.02 s
	1.57 m	1.42 m	1.48 m	1.65 m	
6	2.32 m	2.10 m	2.32 m	2.26 m	
	2.40 m	1.84 m	2.12 m	2.14 m	
7	5.43 t (6.5)	5.12 d (8.0)	5.11 m	5.06 t (8.0)	5.03 s
9	2.18 m	2.20 m	2.18 m	2.19 m	3.02 m
			2.09 m	1.98 m	1.94 dd (5.5, 9.0)
10	1.81 m	1.98 m	1.68 m	1.89 m	4.75 dd (5.5, 6.0)
	1.45 m	1.42 m	1.30 m		
11	3.52 d (10.0)	4.17 d (7.5)	5.11 m	5.12 t (7.0)	4.38 s
13	1.15 m	2.14 m	1.95 m	2.09 m	6.72 t (7.5)
	1.65 m	1.69 m		1.28 m	
14	1.47 m	1.90 m	1.25 m	1.77 m	2.77 m
		1.35 m		1.14 m	2.16 m
15	2.43 m	2.92 m		2.09 m	
16			6.25 s		4.70 s
			5.56 s		4.67 s
17	1.17 d (7.0)	1.28 d (7.5)		1.32 d (7.0)	1.64 s
18	1.08 s	1.25 s	1.04 s	1.39 s	
19	1.68 s	1.51 s	1.58 s	1.56 s	1.39 s
20	1.15 s	1.23 s	1.60 s	1.56 s	
21	3.68 s		3.76 s		3.69 s
22					3.06 s

Compound **2** was isolated as a colorless oil. The HRESIMS of **2** established its molecular formula as C_20_H_34_O_5_, indicating four unsaturations. The ^1^H and ^13^C NMR spectra of **2** were similar to those of capillolide [21], with the exception that the *exo*-methylene proton resonances of the latter were replaced by a methyl doublet at *δ* 1.28 (3H, d, *J* = 7.5 Hz) coupled to a signal at *δ* 2.92. The relative stereochemistry of **2** was deduced mainly by NOESY and by comparison with that found for capillolide. Both H-3 and H-11 showed NOEs with H-1, which further correlated with H-15. Moreover, H_3_-18 shared mutual NOE enhancement with H-3 and H-15. These observations indicated that all H-1, H-3, H-11, H_3_-18, and H-15 were α-oriented with respect to this ring. Furthermore, it was found that the H_3_-20 did not exhibit NOE response with H-11, indicating the β-configuration.

Compound **3** was isolated as a colorless oil. The HRESIMS of **3** established its molecular formula as C_21_H_34_O_4_, indicating five unsaturations. The ^1^H and ^13^C NMR spectra of **3** were similar to those of pseudoplexauric acid methyl ester except for the downfield shifts of C-3 (62.8→71.7), C-4 (60.7→75.2), and C-18 (16.9→23.4) (Table 1) [22], which indicated that two hydroxylated carbons replaced the 3,4-epoxy carbons of the known analogue. This was also supported by the molecular weight of **3**, which was 18 amu higher than that of pseudoplexauric acid methyl ester, as indicated by the HRESIMS data. In the NOESY spectrum of **3**, H-3 showed NOE with H-1, but not with H-18, justifying the assigned relative stereochemistry at C-1, C-3, and C-4.

Compound **4** was isolated as a colorless oil. The HRESIMS of **4** established its molecular formula as C_20_H_32_O_3_, indicating five unsaturations. The ^1^H and ^13^C NMR spectra of **4** were similar to those of 14-deoxycrassin [22,23], with the exception that the *exo*-methylene proton resonances of the latter were replaced by a methyl doublet at *δ* 1.32 (3H, d, *J* = 7.0 Hz) coupled to a signal at *δ* 2.09, which was confirmed by the HMBC experiment. Analyses of NOESY and NMR data revealed that the relative stereochemistry of **4** was the same as 14-deoxycrassin. The relative configuration of the secondary methyl group at C-15 was assigned to be on the same side as the H-1 proton of the *δ*-lactone ring by comparison of ^1^H NMR spectral data with those of dihydrosinularin [*δ* 1.35 (3H, d, *J* = 7 Hz, H_3_-17), 2.2 (1H, m, H-15)] and its 15-epimer [*δ* 1.21 (3H, H_3_-17), 2.80 (1H, m, H-15)] [20,24].

Compound **5** was isolated as a colorless oil. The HRESIMS of **5** established its molecular formula as C_22_H_28_O_8_, indicating nine unsaturations. The^ 1^H and ^13^C NMR spectra of **5** showed great similarity to those of 3α-ethoxyfuranocembrane [25] except that the ethoxyl was replaced by a methoxyl at C-3, and the acetoxy group was replaced by a hydroxy at C-11. The determination of the structure of **5** was further supported by detailed analysis of its 2D NMR data (Figure 2). The relative configuration of **5** was deduced by a NOESY experiment (Figure 3) and by comparison with those of 3α-ethoxyfuranocembranoid [25]. One proton attaching at C-2 and resonating at *δ*_H_ 2.48 was found to show NOE interactions with H-1 and was assigned arbitrarily as H-2α. The isopropenyl group located at C-1 should be β-oriented. NOE correlations between H-5 and H-7, H-7 and H-19 revealed the α-orientation of Me-19, and the *Z* configuration of the double bond Δ^6(7)^. The NOE interaction between H_3_-19 and H_3_-22 indicated the α-orientation of C-3. The trans-arrangement of H-10, and H-11 was implied by the coupling constant *J* (10, 11) ≈ 0 Hz. The NOE interaction between H-11 and H-13 indicated the *Z* configuration of the double bond at Δ^12(13)^ [25]. The 3α-ethoxyfuranocembranoid is an artifact created during isolation by reaction of the solvent ethanol with the natural product danielid [25]. However the danielid and its analogues have not been isolated in our investigation from *Sinularia* sp. In this context, whether compound **5** is created from danielid or its analogues remains to be established.

The identities of compounds **6**–**14** were established by comparison of their spectral data with those of the known compounds reported. They are capilloloid (**6**) [21], sethukarailin (**7**) [26], sinuladiterpenes I (**8**) [27], sinulaflexiolides H (**9**) [28], flexibilisin A (**10**) [29], (1*R*,13*S*,12*S*,9*S*,8*R*,5*S*,4*R*)-9-acetoxy-5,8:12,13-diepoxycembr-15(17)-en-16,4-olide (**11**) [21], 11-*epi*-sinulariolide acetate (**12**) [30], flexibilide (**13**) [21], mandapamate (**14**) [31]. 

Compounds **1**–**14** were evaluated for inhibition of NF-κB activation using the cell-based HEK293 NF-κB luciferase reporter gene assay. The results showed that only **13** exhibited a potent effect with IC_50_ value of 5.30 μg/mL, while other compounds showed only marginal effects. 

## 3. Experimental Section

### 3.1.General Experimental Procedures

The NMR spectra were recorded on a Bruker AC 500 NMR spectrometer with TMS as an internal standard. HR-ESI-MS data were measured on an AQUITY UPLC/Q-TOF micro spectrometer. IR spectra were recorded on a Nicolet 6700 FT-IR spectrometer. Optical rotations were measured on a PerKin Elmer 341 polarimeter using a 1 dm path length cell. ESI-MS data were measured on Bruker’s amaZon SL ion trap LC/MS. Materials for column chromatography were silica gel (Qingdao Marine Chemical Factory, Qingdao, China), Sephadex LH20 (Amersham Pharmacia Biotech AB, Uppsala, Sweden), and YMC Gel ODS-A (YMC, MA, USA). The silica gel GF254 used for TLC was supplied by the Qingdao Marine Chemical Factory, Qingdao, China. HPLC was carried out on SHIMEDZU LC-10ATvp with YMC ODS SERIES.

### 3.2. Animal Material

The soft coral *Sinularia* sp. was collected from Dongluo Island, Hainan province of China in March 2010 (7–10 m depth) and identified by Professor Hui Huang, South China Sea Institute of Oceanology, Chinese Academy of Sciences. A voucher specimen (No. M100301) was deposited in the Key Laboratory of Marine Bio-resources Sustainable Utilization, South China Sea Institute of Oceanology, Chinese Academy of Sciences, Guangzhou, China.

### 3.3. Extraction and Isolation

The soft coral *Sinularia* sp. (7 kg) was extracted three times with 95% EtOH. The extract was concentrated under reduced pressure, and partitioned between H_2_O (4 L) and CHCl_3_ (4 L); the CHCl_3_ layer (101 g) was further partitioned between 85% EtOH (4 L) and petroleum ether (PE; 4 L) to yield 85% EtOH (30 g) and PE (55.3 g) fractions.

The PE extract was subjected to silica gel column chromatography, using a gradient of EtOAc in PE, to give 13 fractions (X1–X13). X8 (2.7 g) was subjected to silica gel column chromatography, using a gradient of EtOAc in PE, to give 7 fractions (X8-1–X8-7). X8-2 was purified by RP HPLC (70% MeOH in H_2_O) to afford **7** (7.2 mg), and **11** (5.2 mg). X8-3 was purified by RP HPLC (70% MeOH in H_2_O) to afford **9** (15.5 mg), and **12** (17.7 mg). X8-4 (430 mg) was further purified on a Sephadex LH20 column to give three subfractions (X8-4-1–X8-4-6). X8-4-2 was purified by RP HPLC (66.5% MeOH in H_2_O) to afford **3** (5.1 mg). X8-4-6 was purified by RP HPLC (70% MeOH in H_2_O) to afford **4 **(15.0 mg). X8-6 was purified by RP HPLC (70% MeOH in H_2_O) to afford **6** (11.7 mg).

The 85% EtOH extract was subjected to silica gel column chromatography, using a gradient of MeOH in CDCl_3_, to give 12 fractions (Y1–Y12). Y3 (1.1 g) was subjected to silica gel column chromatography, using a gradient of EtOAc in PE, to give nine fractions (Y3-1–Y3-9). Y3-3 was further purified on a Sephadex LH20 column to give three subfractions (Y3-3-1–Y3-3-4). Y3-3-4 was purified by RP HPLC (66.5% MeOH in H_2_O) to afford **5** (5.1 mg), and **10** (11.1 mg). Y3-7 was purified by RP HPLC (66.5% MeOH in H_2_O) to afford **13** (5.0 mg), and **14** (4.8 mg). Y4 (1.2 g) was subjected to silica gel column chromatography, using a gradient of EtOAc in PE, to give six fractions (Y4-1–Y4-6). Y4-1 was purified by RP HPLC (55% MeOH in H_2_O) to afford **2** (17.2 mg). Y4-3 was purified by RP HPLC (55% MeOH in H_2_O) to afford **1** (4.3 mg), and **8** (3.9 mg).

Sinuflexibilin A (**1**): colorless oil; [α]_D_^25^ = +16.7 (*c* = 0.03, CHCl_3_); IR (KBr) ν_max_ 3364, 2967, 1709, 1650, 1453 cm^−^^1^; ^1^H and ^13^C NMR in Table 1 and Table 2; ESIMS *m*/*z* 409 [M + Na]^+^, 795 [2M + Na]^+^, HRESIMS *m*/*z* 409.2574 (calcd for C_21_H_38_O_6_Na, 409.2566). 

Sinuflexibilin B (**2**): colorless oil; [α]_D_^25^ = +23.0 (*c* = 0.10, CHCl_3_); IR (KBr) ν_max_ 3440, 2937, 1687, 1465 cm^−^^1^; ^1^H and ^13^C NMR in Table 1 and Table 2; HRESIMS *m*/*z* 377.2015 (calcd for C_20_H_34_O_5_Na, 377.2018).

Sinuflexibilin C (**3**): colorless oil; [α]_D_^25^ = +5.0 (*c* = 0.01, CHCl_3_); IR (KBr) ν_max_ 3428, 2928, 1720, 1439 cm^−^^1^; ^1^H and ^13^C NMR in Table 1 and Table 2; HRESIMS *m*/*z* 373.2231 (calcd for C_21_H_34_O_4_Na, 373.2202).

Sinuflexibilin D (**4**): colorless oil; [α]_D_^25^ = +6.0 (*c* = 0.01, CHCl_3_); IR (KBr) ν_max_ 3462, 2933, 1725, 1437 cm^−^^1^; ^1^H and ^13^C NMR in Table 1 and Table 2; HRESIMS *m*/*z* 343.2244 (calcd for C_20_H_32_O_3_Na, 343.2249).

Sinuflexibilin E (**5**): colorless oil; [α]_D_^25^ = +23.3 (*c* = 0.03, CHCl_3_); IR (KBr) ν_max_ 3349, 2922, 1751, 1722, 1436 cm^−^^1^; ^1^H and ^13^C NMR in Table 1 and Table 2; HRESIMS *m*/*z* 443.1667 (calcd for C_22_H_28_O_8_Na, 443.1682 ).

### 3.4. The Cell-Based HEK293 NF-κB Luciferase Reporter Gene Assay

All compounds were evaluated for inhibition of NF-κB activation using the cell-based HEK 293 NF-κB luciferase reporter gene assay according to the previously reported procedures [19]. 

## 4. Conclusions

The investigation of bioactive natural products from a Hainan soft coral, *Sinularia* sp., has led to the isolation of five new cembranes, sinuflexibilins A–E (**1**–**5**), along with nine other known diterpenoids (**6**–**14**). Compound **13** exhibited significant inhibition activity of NF-κB activation using the cell-based HEK293 NF-κB luciferase reporter gene assay with an IC_50_ of 5.30 μg/mL.

## Supplementary Files

Supplementary File 1: PDF-Document (PDF, 504 KB)
