# Supplementary material for: New Cembrane Diterpenoids from a Hainan Soft Coral Sinularia sp
Source: Mar Drugs. 2012 Sep 18;10(9):2023–32. doi: 10.3390/md10092023 (PMC3475270; doi:10.3390/md10092023)

Supplementary Information

Table of Contents

Figure S1. ^{1}H NMR Spectrum (500 MHz) of compound **1** in MeOD

Figure S2. ^{13}C NMR Spectrum (125 MHz) of compound **1** in MeOD

Figure S3. ^{1}H NMR Spectrum (500 MHz) of compound **2** in MeOD

Figure S4. ^{13}C NMR Spectrum (125 MHz) of compound **2** in MeOD

Figure S5. ^{1}H NMR Spectrum (500 MHz) of compound **3** in $CDCl_3$

Figure S6. ^{13}C NMR Spectrum (125 MHz) of compound **3** in $CDCl_3$

Figure S7. ^{1}H NMR Spectrum (500 MHz) of compound **4** in $CDCl_3$

Figure S8. ^{13}C NMR Spectrum (125 MHz) of compound **4** in $CDCl_3$

Figure S9. ^{1}H NMR Spectrum (500 MHz) of compound **5** in $CDCl_3$

Figure S1. ^{1}H NMR Spectrum (500 MHz) of compound **1** in MeOD.

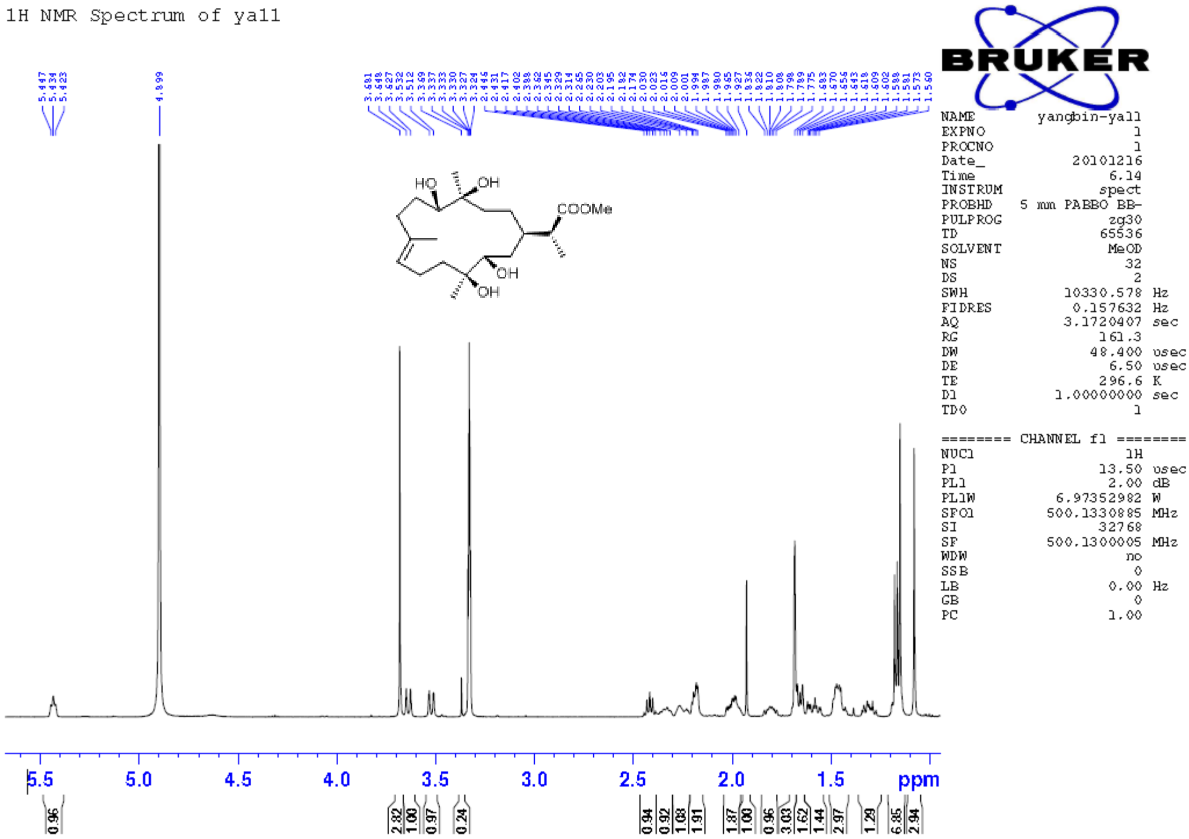

Figure S2. ^{13}C NMR Spectrum (125 MHz) of compound **1** in MeOD.

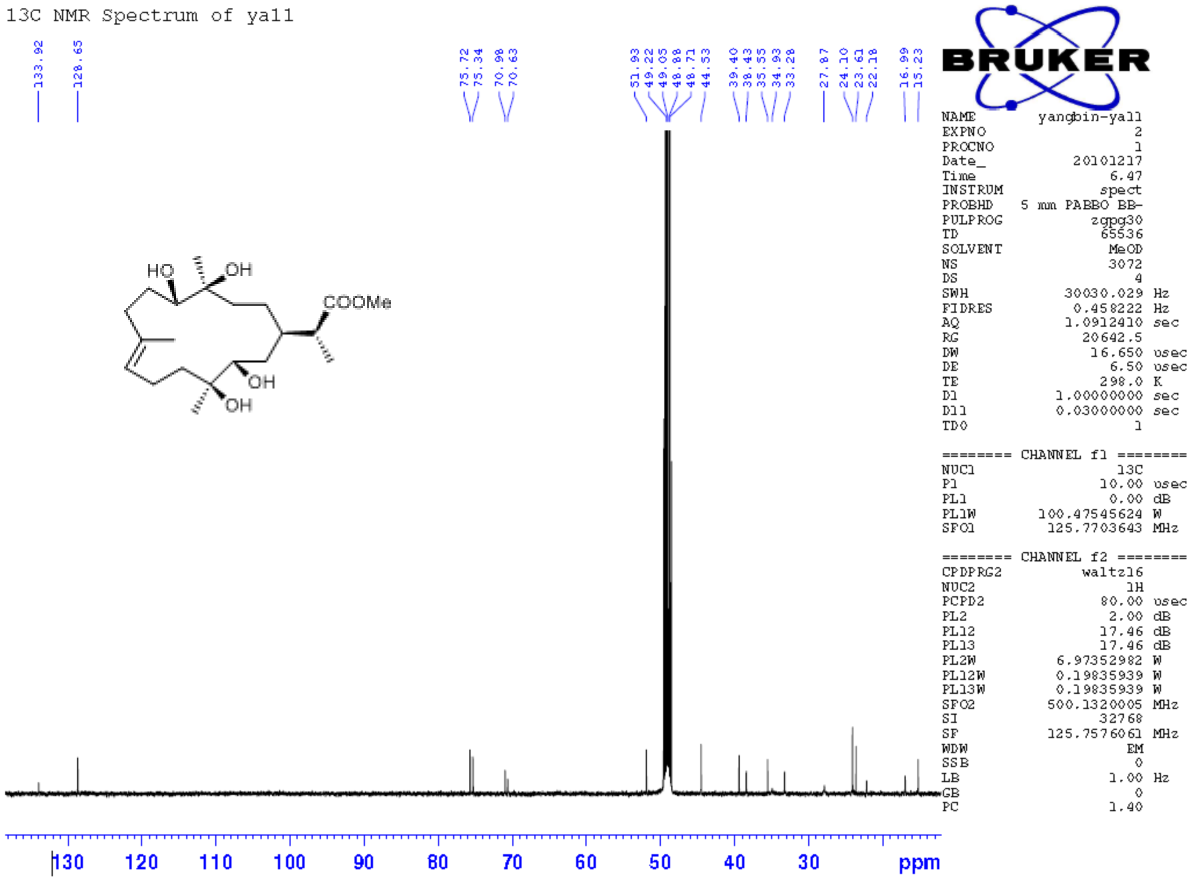

Figure S3. 1H NMR Spectrum (500 MHz) of compound **2** in MeOD.

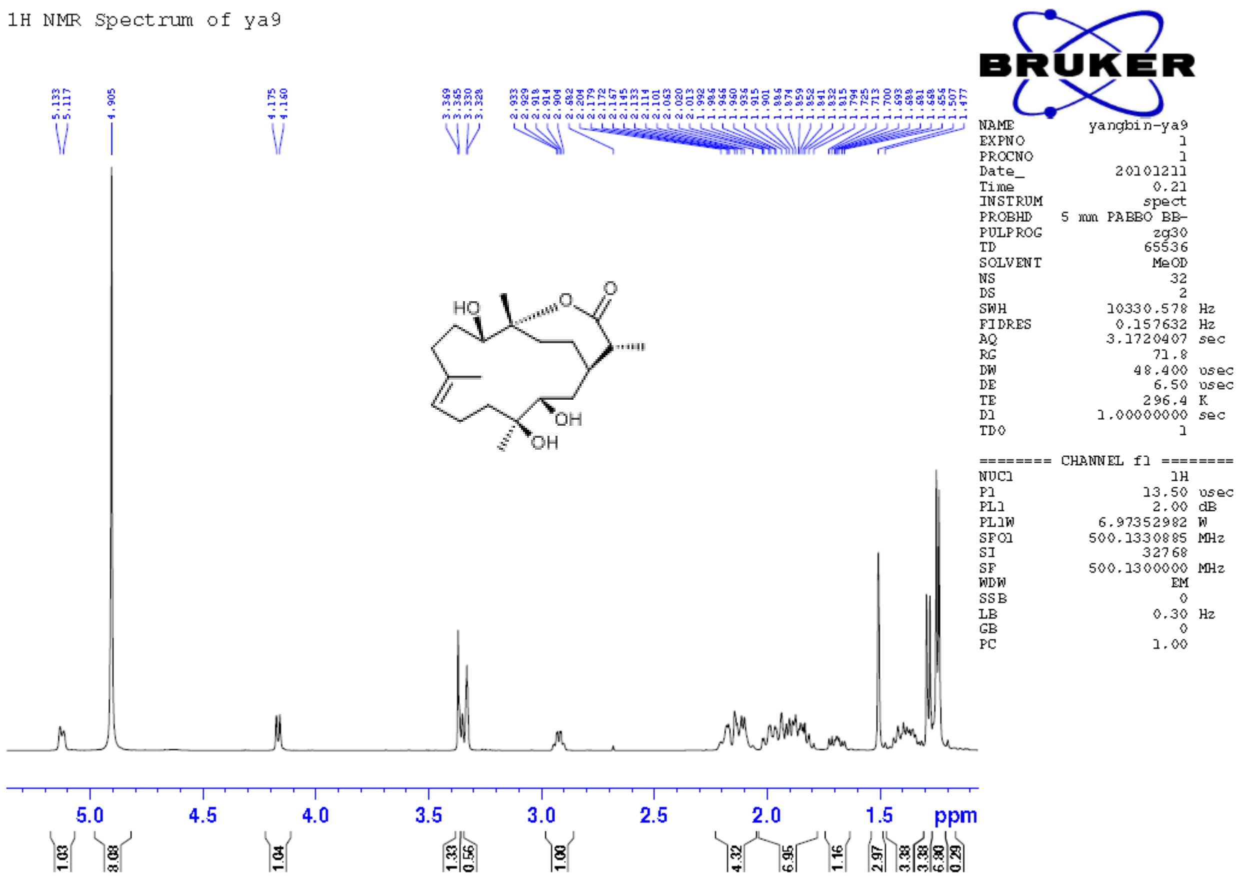

Figure S4. 13C NMR Spectrum (125 MHz) of compound **2** in MeOD.

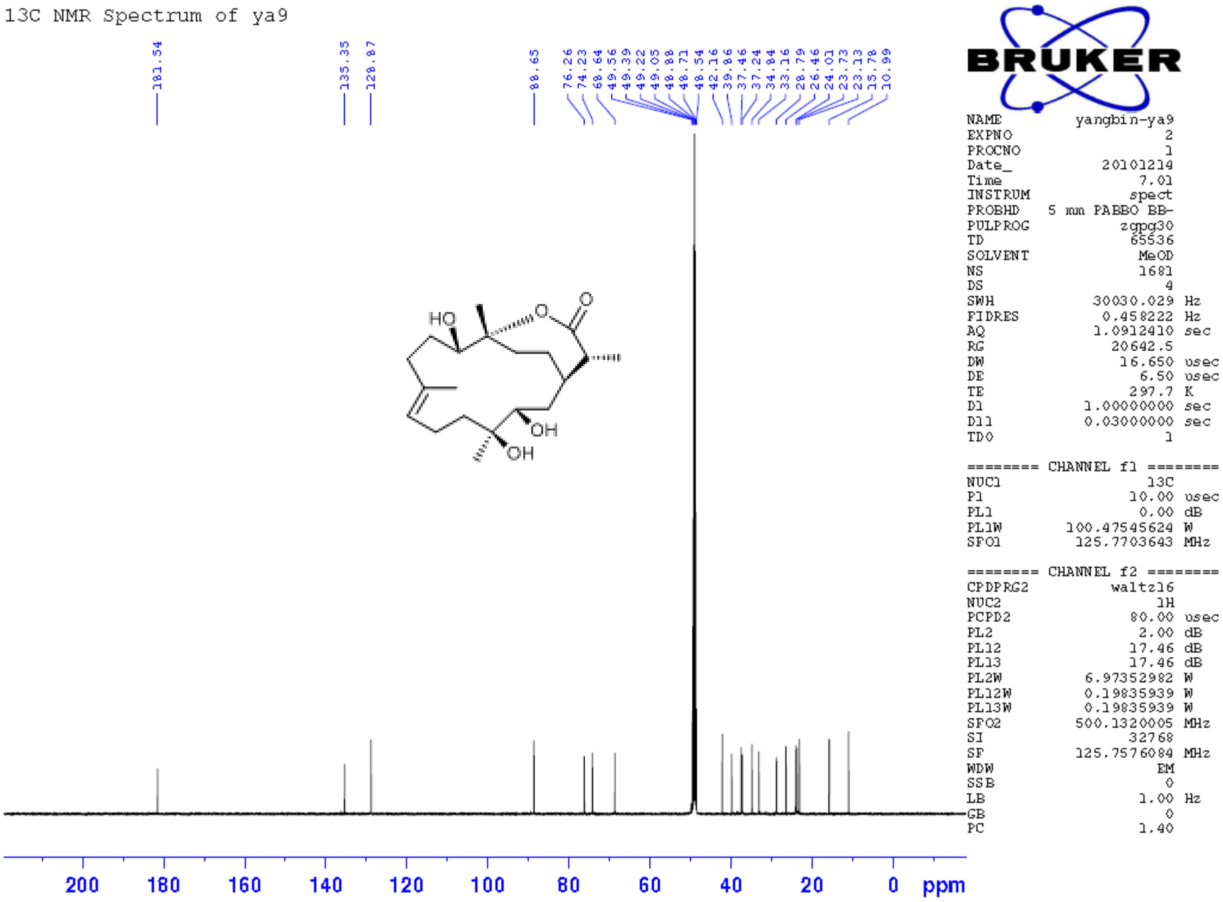

Figure S5. 1H NMR Spectrum (500 MHz) of compound **3** in CDCl3.

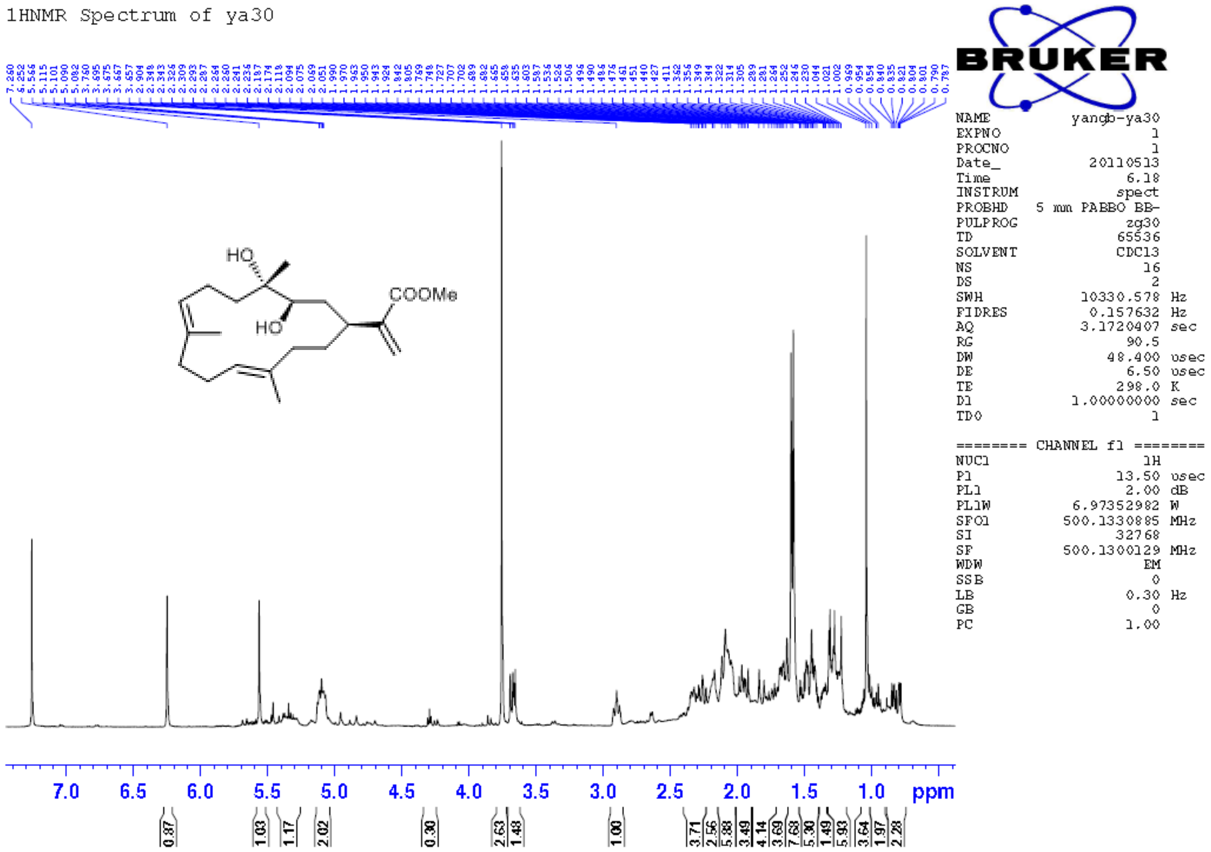

Figure S6. 13C NMR Spectrum (125 MHz) of compound **3** in CDCl3.

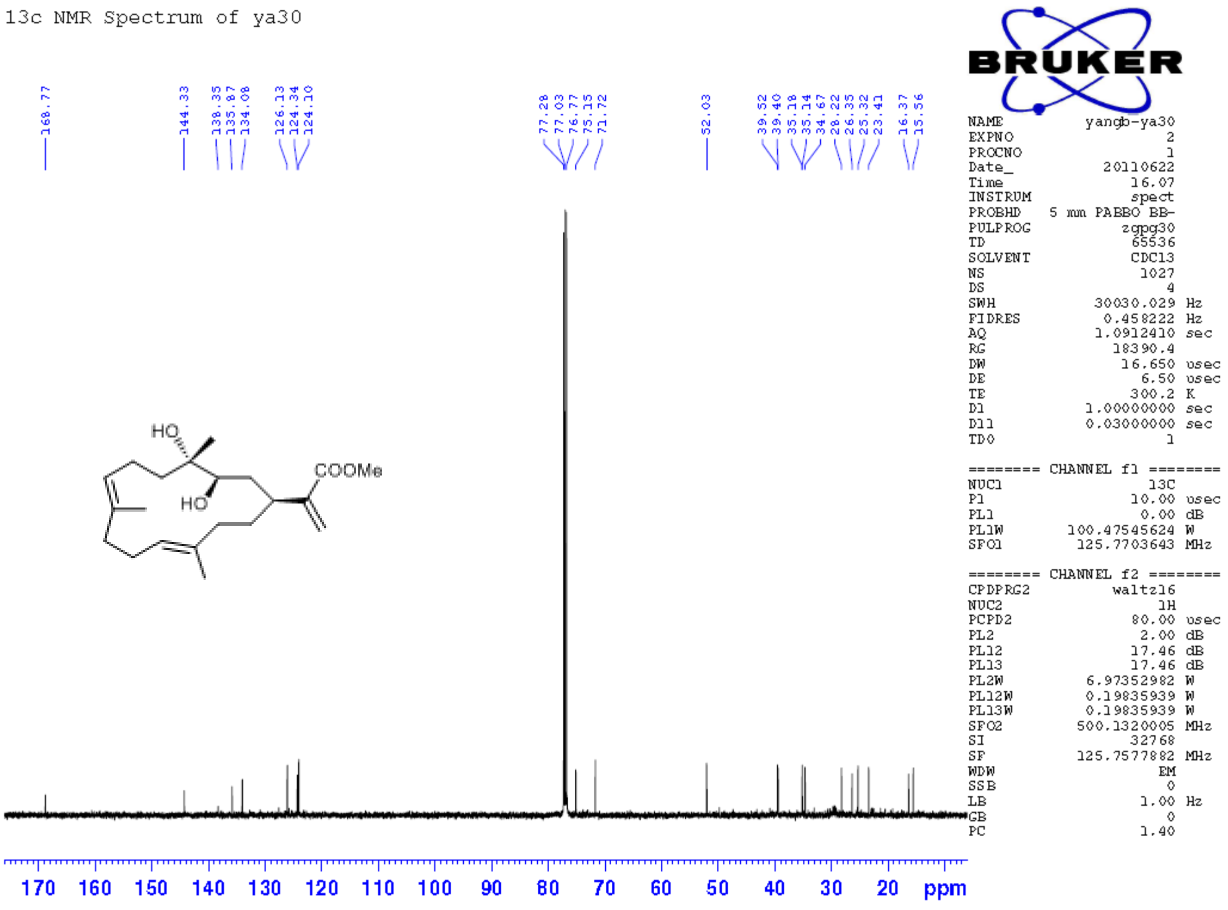

Figure S7. 1H NMR Spectrum (500 MHz) of compound **4** in CDCl3.

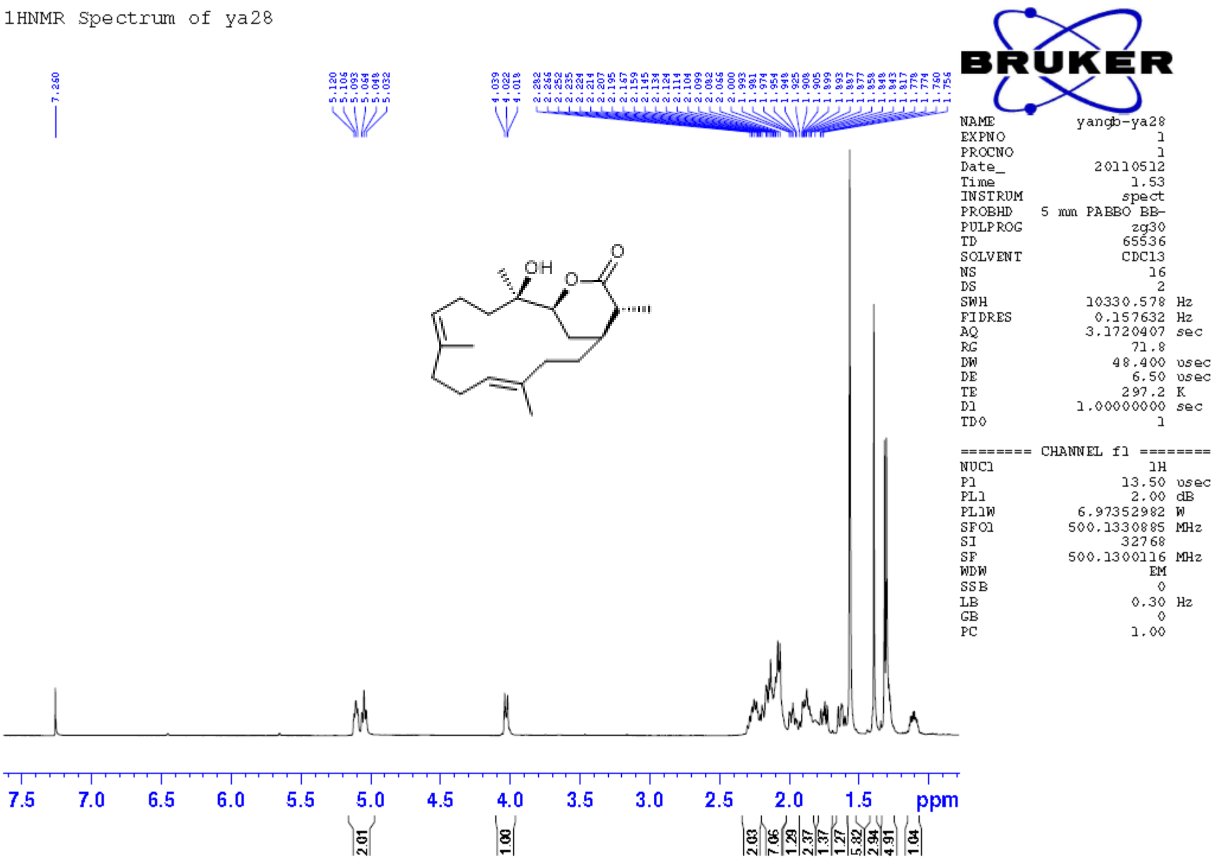

Figure S8. 13C NMR Spectrum (125 MHz) of compound **4** in CDCl3.

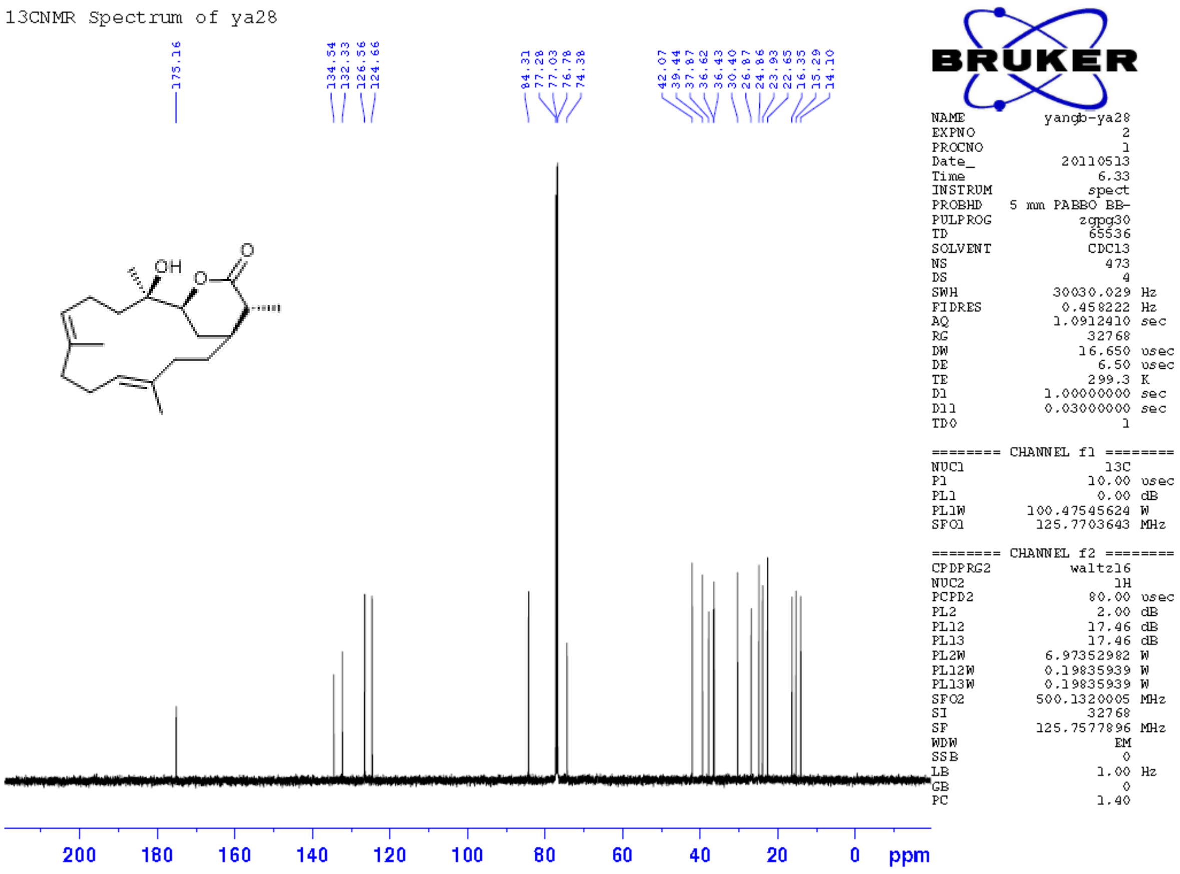

Figure S9. 1H NMR Spectrum (500 MHz) of compound **5** in CDCl3.

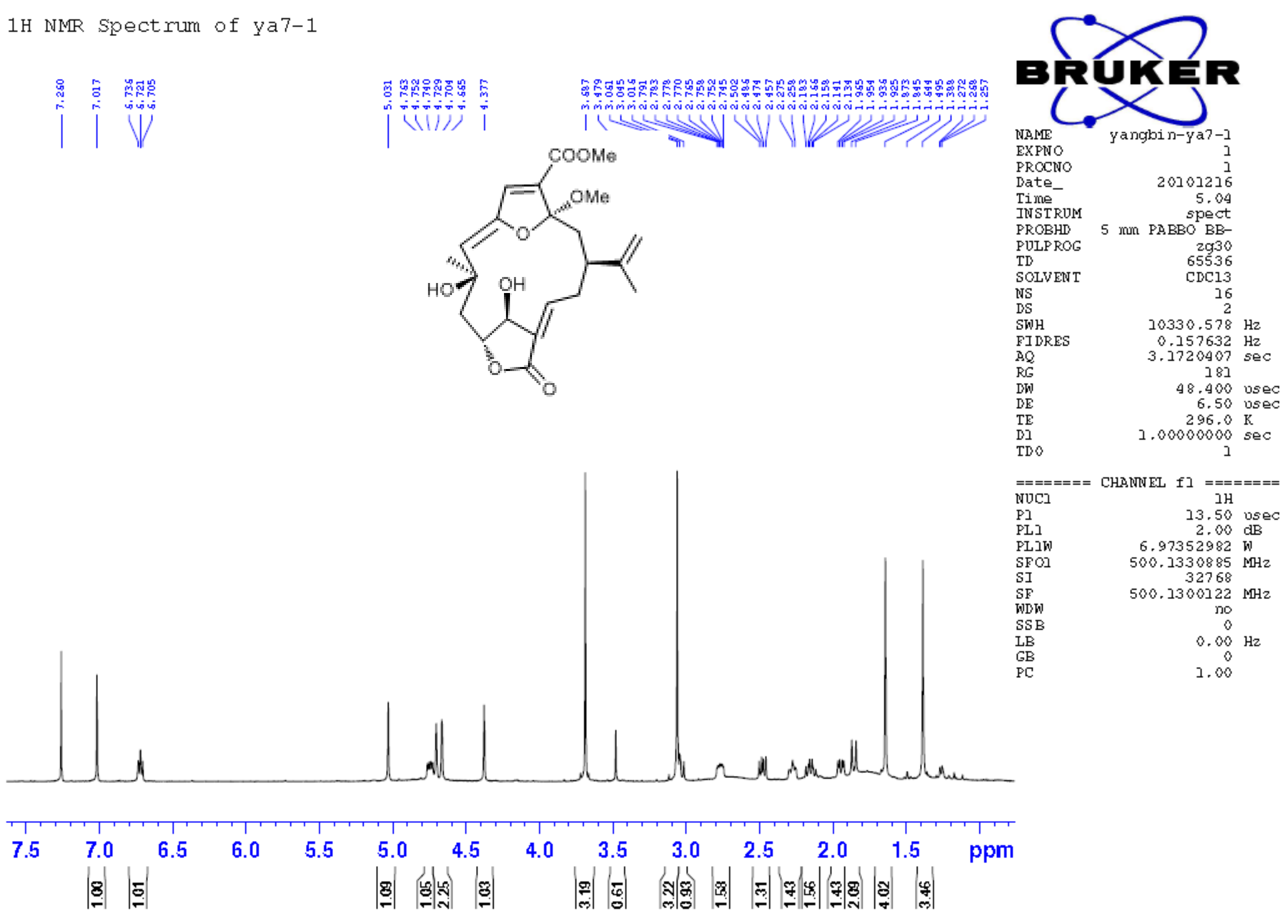

Figure S10. 13C NMR Spectrum (125 MHz) of compound **5** in CDCl3.

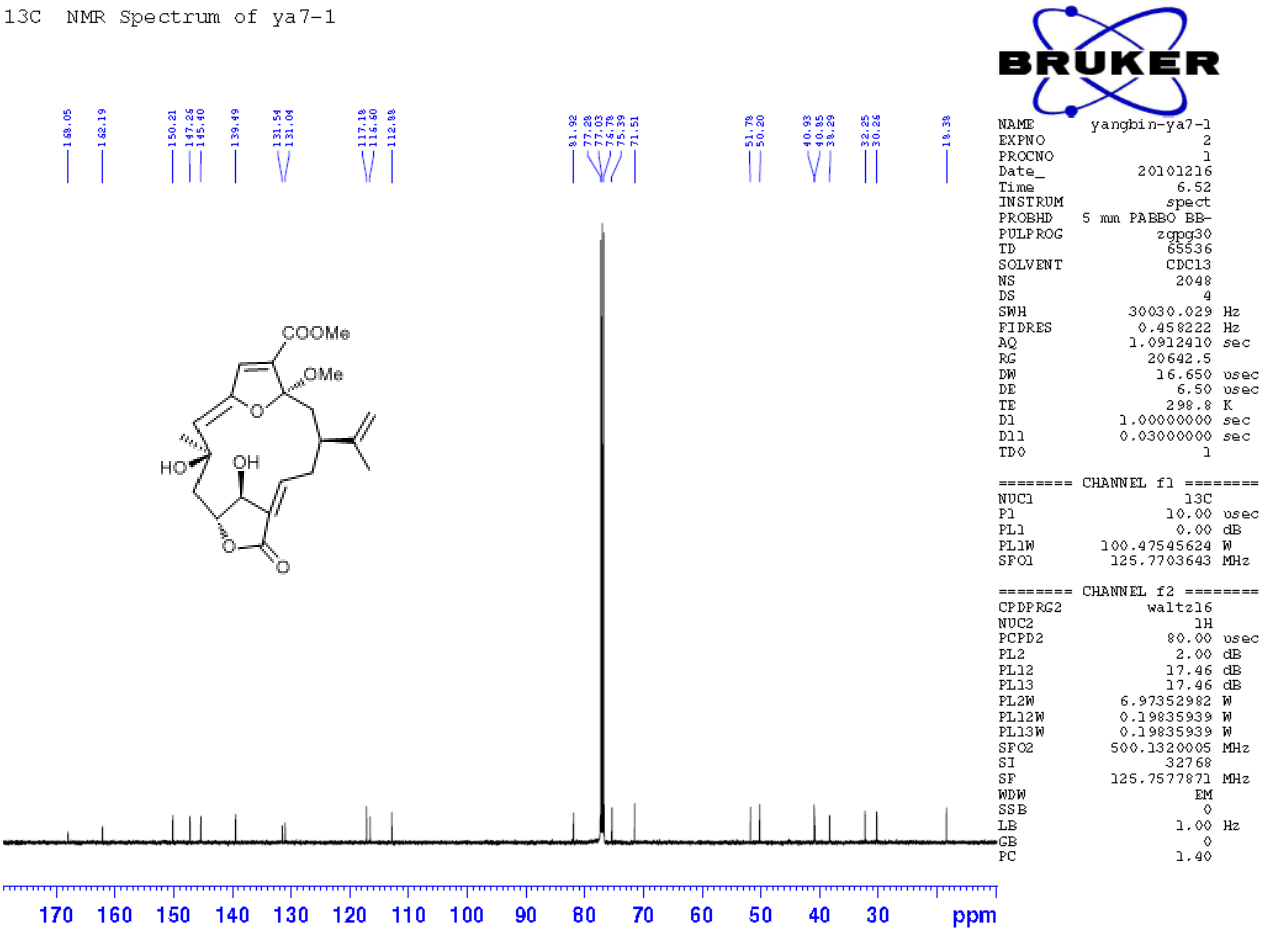

Supplement: Supplementary File 1: — PDF-Document (PDF, 504 KB) [file marinedrugs-10-02023-s001.pdf]
